# Salient Findings on Host Range, Resistance Screening, and Molecular Studies on Sterility Mosaic Disease of Pigeonpea Induced by *Pigeonpea sterility mosaic viruses* (*PPSMV-I* and *PPSMV-II*)

**DOI:** 10.3389/fmicb.2022.838047

**Published:** 2022-04-01

**Authors:** B. R. Sayiprathap, A. K. Patibanda, V. Prasanna Kumari, K. Jayalalitha, H. K. Ramappa, E. Rajeswari, L. Karthiba, K. Saratbabu, Mamta Sharma, H. K. Sudini

**Affiliations:** ^1^International Crops Research Institute for the Semi-Arid Tropics, Hyderabad, India; ^2^Department of Plant Pathology, Acharya N. G. Ranga Agricultural University, Guntur, India; ^3^Department of Crop Physiology, Acharya N. G. Ranga Agricultural University, Guntur, India; ^4^Department of Plant Pathology, University of Agricultural Sciences, Bengaluru, India; ^5^Department of Plant Pathology, Tamil Nadu Agricultural University, Coimbatore, India

**Keywords:** *Cajanus cajan*, SMD, *PPSMV-I*, *PPSMV-II*, host-range, host-plant resistance (HPR)

## Abstract

Two distinct emaraviruses, *Pigeonpea sterility mosaic virus-I* (*PPSMV-I*) and *Pigeonpea sterility mosaic virus-II* (*PPSMV-II*) were found to be associated with sterility mosaic disease (SMD) of pigeonpea [*Cajanus cajan* (L.) Millsp.]. The host range of both these viruses and their vector are narrow, confined to *Nicotiana benthamiana* identified through mechanical transmission, and to *Phaseolus vulgaris* cvs. Top Crop, Kintoki, and Bountiful (F: Fabaceae) through mite transmission. A weed host *Chrozophora rottleri* (F: Euphorbiaceae) was also infected and tested positive for both the viruses in RT-PCR. Among the wild *Cajanus* species tested, *Cajanus platycarpus* accessions 15661, 15668, and 15671, and *Cajanus scarabaeoides* accessions 15683, 15686, and 15922 were infected by both the viruses and mite vector suggesting possible sources of SMD inoculum. Though accession 15666 of *C. platycarpus*, 15696 of *C. scarabaeoides*, and 15639 of *Cajanus lanceolatus* were infected by both the viruses, no mite infestation was observed on them. Phylogenetic analysis of nucleotide sequences of RNA-1 and RNA-2 of *PPSMV-I* and *PPSMV-II* isolates in southern India revealed significant divergence especially *PPSMV-II*, which is closely related to the *Fig mosaic virus* (*FMV*) than *PPSMV-I*. In multilocation testing of pigeonpea genotypes for their broad-based resistance to SMD for two consecutive years, genotypes ICPL-16086 and ICPL-16087 showed resistance reaction (<10% incidence) in all three locations studied. Overall, the present study gives a clear idea about the host range of *PPSMV-I* and *PPSMV-II*, their molecular relationship, and sources of resistance. This information is critical for the development of reliable diagnostic tools and improved disease management strategies.

## Introduction

Sterility mosaic disease (SMD) is one of the important production constraints of pigeonpea cultivation in the Indian subcontinent (Mitra, 1931; Reddy et al., 1998). SMD is caused by two distinct emaraviruses, *Pigeonpea sterility mosaic virus-I* (*PPSMV-I*) and *Pigeonpea sterility mosaic virus-II* (*PPSMV-II*; Elbeaino et al., 2014, 2015; Kumar et al., 2017; Patil et al., 2017; Sayiprathap et al., 2020) and transmitted by an eriophyid mite *Aceria cajani* Channabasavanna (Acari: Arthropoda) in a semi-persistence manner (Kulkarni et al., 2002). SMD symptoms include yellow mosaic or chlorotic ring spots, reduced leaf size, stunting, excessive vegetative growth, and partial-to-complete cessation of flowering (sterility). Generally, the nature and severity of symptoms depend on the host genotype and stage of infection (Jones et al., 2004; Patil and Kumar, 2015).

Though SMD was first reported in 1931 from the Bihar state of India (Mitra, 1931), its etiology remains a mystery for several decades until Kumar et al. (1999; 2000; 2001a) unfolded the causal agent for SMD of pigeonpea as *Pigeonpea sterility mosaic virus* (*PPSMV*, later renamed as *PPSMV-I*), a putative RNA virus of negative orientation. Complete genome sequences of *PPSMV-I* was reported to contain four to five RNA segments (RNA-1 to RNA-5) and *PPSMV-II* with six RNA segments (RNA-1 to RNA-6) have been published recently (Elbeaino et al., 2015; Kumar et al., 2017; Patil et al., 2017). Based on the genome organization and morphological features, both the viruses were taxonomically included in the genus *Emaravirus* in the recently created family *Fimoviridae* of order *Bunyavirales* (Elbeaino et al., 2018). Preliminary field observations suggest that *PPSMV-I* was associated with chlorotic ring spots and line patterns, whereas *PPSMV-II* induces leaf mosaic, stunting, and sterility symptoms. A more severe form of SMD was shown by plants with mixed infection of both the viruses and more frequently occurs in nature (Elbeaino et al., 2015; Patil et al., 2017). The vector, eriophyid mite, is host specific with a narrow host range confined to pigeonpea and few of its wild relatives (Kumar et al., 2003). It is the sole vector responsible for the transmission of SMD in pigeonpea (Seth, 1962; Reddy et al., 1998; Kulkarni et al., 2002; Jones et al., 2004; Kumar et al., 2004; Patil and Kumar, 2015). Though few alternative hosts of *PPSMV* have been reported, these hosts are not congenial for vector multiplication (Kumar et al., 2002a; Kulkarni et al., 2003a). The source of resistance to SMD was first reported in a pigeonpea landrace, “Sabour 2E” in India (Alam, 1933). Subsequently, several disease-resistant and tolerant lines were identified. Efforts on identifying the sources of resistance to SMD were initiated at ICRISAT in 1975. Over 13,015 pigeonpea accessions from the global pigeonpea germplasm collection at ICRISAT were screened for SMD, and 326 resistant and 97 tolerant lines were reported (Nene, 1995). Recently, 28 pigeonpea genotypes have been identified as resistant to SMD from a preliminary screening of 976 pigeonpea accessions evaluated at eight different geographical locations in India (Sharma et al., 2015).

Studies on the host ranges of *PPSMV-I* and *PPSMV-II* help us develop better management strategies; however, there is little knowledge about the host range of *PPSMV* and its vector. In a previous study, when there was no information about the two distinct emaraviruses associated with SMD, an attempt was made to study the natural and experimental host range for *PPSMV* and concluded that a couple of *Chrozophora rottleri* weed plants tested positive for *PPSMV* (Kulkarni et al., 2003a). A thorough understanding of the genetic variability of emaraviruses associated with SMD of pigeonpea is essential for the development of reliable and robust diagnostic tools (Kallinen et al., 2009; Walia et al., 2009; Dong et al., 2016; Stewart, 2016). Host-plant resistance is the most viable, realistic, and cost-effective option for the management of any viral disease. However, developing stable resistant varieties of pigeonpea is complicated due to the genetic flexibility of the pathogen, which is affected by location-specific environments (Nene et al., 1989; Amin et al., 1993; Sharma and Pande, 2011; Sharma et al., 2012, 2015). By keeping the above fact in view, we studied a wide range of crop and weed species, including wild *Cajanus* accessions, to identify the host range of *PPSMV*. The SMD samples collected from different geographical locations in southern India were analyzed for their molecular relationship between *PPSMV-I* and *PPSMV-II*. Furthermore, several pigeonpea genotypes were screened at three distinct geographical locations for two consecutive years in order to identify their broad-based resistance to SMD.

## Materials and Methods

### *Pigeonpea sterility mosaic virus* Inoculum

*Pigeonpea sterility mosaic virus* culture was maintained on susceptible pigeonpea cultivar ICP 8863 (Maruti) in a glasshouse at 27 ± 1°C with 70–80% relative humidity. The leaf stapling technique (Nene and Reddy, 1976) was used to inoculate 12- to 15-day-old healthy pigeonpea seedlings.

### Transmission of *Pigeonpea sterility mosaic virus*

#### Mechanical Sap Inoculation

Sterility mosaic disease-infected young leaf tissue was ground in 0.05 M phosphate buffer (1:10 w/v) containing 0.1% β-mercaptoethanol (β-ME) using mortar and pestle on an ice bucket, filtered through a muslin cloth, and inoculated immediately onto a test plant at the two- to three-leaf stage by dusting celite (abrasive) (Sigma-Aldrich) with the forefinger. The inoculated leaves were then slowly rinsed with distilled water and kept in a vector-proof glasshouse at 27 ± 1°C.

#### Leaf-Stapling Method

Leaf-stapling method of transmission of *PPSMV* was followed as per the protocol described by Nene and Reddy (1976). Young SMD-infected leaflets collected in a moist cloth bag were observed for mite infestation under a binocular microscope to ensure a minimum of 10 mites per leaf. The mite-infested leaflets were then stapled onto test plants at the two- to three-leaf stage in such a way that the undersurface of the diseased leaflet comes in contact with both surfaces of the leaf of the test plant to anchor mites for transfer and their feeding results in *PPSMV* transmission onto the test plant.

### Direct Antigen Coating-ELISA

Polyclonal antibodies to *PPSMV* were developed at ICRISAT, Hyderabad, and were used to detect the virus in plant tissues by direct antigen coating (DAC)-ELISA as per the protocol suggested by Kumar et al. (2003). To minimize the non-specific reactions to host plant antigen, polyclonal antisera were cross-absorbed in healthy pigeonpea (cv. ICP 8863) leaf sap at 10 mg/ml in phosphate-buffered saline containing 0.2% ovalbumin and 2% PVP, at 37°C for 45 min. DAC-ELISA was performed by grinding a leaf sample in carbonate buffer, pH 9.6 (1:10, w/v), and the extract was added to wells of MaxiSorp ELISA plates (Nunc, Thermo Fisher Scientific, Denmark). The cross-absorbed polyclonal antiserum was used at 1:3,000 dilution. Alkaline phosphatase (ALP)-labeled goat anti-rabbit IgG (Sigma) was used at 1:4,000 dilution for detecting the immobilized antigen–antibody complex, and p-nitrophenylphosphate (Sigma) (0.5 mg/ml in 10% diethanolamine buffer, pH 9.8) was added as the substrate. The plate was observed for color changes and recorded as weak positive for light yellow and strongly positive for deep yellow.

### Total RNA Extraction and Reverse Transcription-Polymerase Chain Reaction

Briefly, 100 mg of leaf tissue was ground in liquid nitrogen to a fine powder. Total RNA was extracted using the QIAGEN RNeasy plant mini kit by following the manufacturer’s instructions. The RNA quantity and quality were assessed using a spectrophotometer (NanoDrop 8000, Thermo Fisher Scientific) and stored in a refrigerator at −20°C. RT-PCR was performed as per the protocol suggested by Elbeaino et al. (2015). Total RNA (500 ng) was randomly reverse transcribed by adding 4 μl of 5× M-MuLV buffer (New England Biolabs, Ipswich, MA, United States), 0.5 μl of 10 mM dNTPs, 2 μl of 10 mM DTT, 250 ng of random primer, and 200 U of M-MuLV reverse transcriptase (New England Biolabs, Ipswich, MA, United States) in a final volume of 20 μl for 1 h at 39°C followed by inactivation of the enzyme at 65°C for 20 min. Synthetic oligonucleotide primers (Table 1) were used to amplify RNA segments *PPSMV-I* and *PPSMV-II*. Random-primed cDNA (2 μl) was added to 5× Taq polymerase buffer (New England Biolabs, Ipswich, MA, United States) containing MgCl_2_ to a final concentration of 1 mM, 0.2 mM dNTPs, 0.2 μM of each specific primer, and 1 U of Taq DNA polymerase in a final volume of 25 μl. The PCR mixture tube was incubated by 1 cycle of denaturation at 94°C for 4 min, followed by 35 cycles at 92°C for 30 s, 45–65°C for 30 s, and 72°C for 30 s. The final extension was at 72°C for 7 min. The amplification products were resolved in a 1.2% TBE agarose gel, visualized, and documented by a gel–doc system (Major Science image analyzer).

**TABLE 1:** Synthetic oligonucleotide primers used for polymerase chain reaction (PCR) amplification.

Virus	Segment	Primer sequence (5′—3′)	Amplicon size
*Pigeonpea sterility mosaic virus I* (*PPSMV-I*)	RNA-1	ATCTAGGTGGTGTGTTTGACA	322 bp
		AACTTGCTCAAAATTCTCAAGC	
	RNA-2	GATGGTCTAGTAATTAGTTTGAG	392 bp
		CTCTATGTGCTTATGTCCAGCA	
	RNA-3	ACATAGTTCAATCCTTGAGTGCG	322 bp
		ATATTTTAATACACTGATAGGA	
*Pigeonpea sterility mosaic virus II* (*PPSMV-II*)	RNA-1	ATCAATACTCCATAGTGCACCT	332 bp
		ACACCAACAGAAATATTCTTGGTG	
	RNA-2	GACTTACATGATTATTGCTCCA	384 bp
		TGTCATATGATCACTATCTGTA	
	RNA-3	GAGAGTAGTGAGTTGGAACCGAT	284 bp
		GAGTATCCCAGCAGCCATTATT	

### Determining Host Range

Seeds of 11 *Nicotiana* species obtained from the Central Tobacco Research Institute (CTRI), Rajamahendravaram, Andhra Pradesh, India, were used in the study along with five herbaceous plants, such as two pigeonpea cultivars (ICP-8863 and ICP-2376), *Phaseolus vulgaris*, *Vigna unguiculata* c-152, and *Chenopodium album*, and were grown in 8-inch pots and inoculated mechanically with SMD-infected sap and kept inside a glasshouse at 27 ± 1°C. In another set, seeds of 24 accessions of 12 *Cajanus* species obtained from the gene bank of ICRISAT, India, were scarified by slicing the seed coat with a scalpel blade, treated with thiram at 30 mg/10 g of seeds, and were sown in 8-inch pots along with 16 cultivated species intercropped with pigeonpea and 46 weed species raised to the two- to three-leaf stage and inoculated by leaf stapling method and kept in the glasshouse at 27 ± 1°C with 70–80% relative humidity. The test plants were monitored regularly for symptom appearance and tested for the infection of *PPSMV* by DAC-ELISA. The ELISA-positive samples were tested further for the infection of *PPSMV-I* and/or *PPSMV-II* using oligonucleotide primers corresponding to the RNA-3 segment through RT-PCR.

### Molecular Variability in *Pigeonpea sterility mosaic virus I* and *II* Isolates

#### Survey and Sample Collection

Pigeonpea leaves exhibiting typical SMD symptoms were collected during a roving survey conducted in 2017/2018 from different geographical locations covering Andhra Pradesh, Karnataka, Tamil Nadu, and Telangana states in southern India. The collected samples were placed in ziplock plastic bags and transported in cold packs to ICRISAT, Hyderabad, snap frozen in liquid nitrogen, and stored in a −80°C freezer.

#### Total RNA Extraction, Reverse Transcription-Polymerase Chain Reaction, Sequencing, and Phylogenetic Analysis

Total RNA was extracted using the QIAGEN RNeasy plant mini kit by following the manufacturer’s instructions. RT-PCR was performed using oligonucleotide primers (Table 1) to amplify partial RNA-1 and RNA-2 of *PPSMV-I* and *PPSMV-II*. Amplicons were purified and sequenced by Sanger’s dideoxy chain-termination method [ABI 3730 (48 capillaries) electrophoresis]. Later, the nucleotide homology searches were done with the BLASTN sequence analysis of the NCBI^1^. Multiple alignments were performed using MUSCLE (Edgar, 2004), and the phylogenetic tree was constructed by MEGA X (Kumar et al., 2018) employing maximum-likelihood (ML) criterion using the neighbor-joining method, to examine the molecular relationship between and among the isolates of *PPSMV-I* and *PPSMV-II* in southern India.

### Multi-Environment Evaluation of Pigeonpea Genotypes for Their Reaction to Sterility Mosaic Disease

Twenty pigeonpea advanced breeding lines along with SMD-susceptible genotype, ICP 8863 (Maruti), were obtained from the Pigeonpea Breeding Unit, ICRISAT, Patancheru, India. All these genotypes were evaluated for their reaction to SMD at three geographical locations, such as Bengaluru (13°04′48″N 77°34′14″E, altitude-914 m), Coimbatore (11°01′24″N 76°55′45″E, altitude-431 m), and Patancheru (17°30′35″N 78°16′31″E, altitude-547 m), in southern India for two consecutive years (rainy season 2017/2018 and 2018/2019). Plants were raised to the two- to three-leaf stage and inoculated by following the leaf stapling method as described earlier.

### Data Collection and Analysis

The test genotypes were regularly monitored for the symptom expression, SMD incidence was recorded, and percent disease incidence was calculated using the formula:


$$\%\mathrm{SMD}\mathrm{incidence}=\frac{\mathrm{Number}\mathrm{of}\mathrm{SMD}\mathrm{infected}\mathrm{plants}}{\mathrm{Total}\mathrm{number}\mathrm{of}\mathrm{plants}}\times 100$$

Based on the SMD incidence, test genotypes were categorized as resistant (≤10.0% incidence), moderately resistant (10.1–20.0% incidence), susceptible (20.1–40.0% incidence), and highly susceptible (>40.0% incidence) (Sharma et al., 2015).

## Results

### Host Range of *Pigeonpea sterility mosaic virus*

**FIGURE 1:**
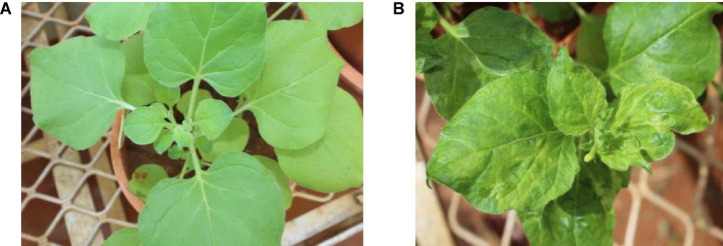
Experimental host range of *Pigeonpea sterility mosaic virus* (*PPSMV*). Healthy *Nicotiana benthamiana* **(A)**, sap inoculated *PPSMV* affected *N. benthamiana* exhibiting yellow mosaic symptoms **(B)**.

**FIGURE 2:**
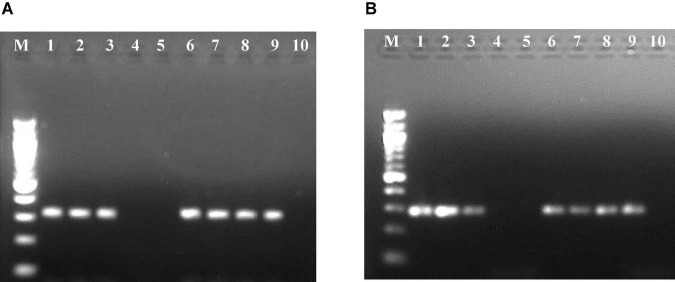
Resolution of reverse transcription-polymerase chain reaction (RT-PCR) products of RNA-3 segment of *Pigeonpea sterility mosaic virus I* (*PPSMV-I*) **(A)** and *Pigeonpea sterility mosaic virus II* (*PPSMV-II*) **(B)** in 1.2% agarose gels. Standard 100-bp DNA marker (lane M), pigeonpea ICP 8863 (lane 1), pigeonpea ICP 2376 (lane 2), *Nicotiana benthamiana* (lane 3), *N. clevelandii* (lane 4), *Macroptilium atropurpureum* (lane 5), *Phaseolus vulgaris* cv. Top Crop (lane 6), *P. v.* cv. Bountiful (lane 7), *P. v.* cv. Kintoki (lane 8), *Chrozophora rottleri* (lane 9), *Hibiscus panduriformis* (lane 10).

Of the 11 *Nicotiana* species (Table 2) and five herbaceous plants tested by mechanical sap inoculation, only *Nicotiana benthamiana* was found positive for *PPSMV* infection. Symptoms appeared after 35–40 days of post-inoculation (dpi) as chlorotic spots, deformation of leaves, and stunted growth, while in the advanced stage (65–70 dpi), the symptoms were systemic and appeared on young leaves as yellow mosaic and crinkled leaves (Figure 1). However, the *PPSMV* was not mechanically transmitted onto the pigeonpea. The ELISA-positive samples when tested in RT-PCR were found infected with both the viruses (*PPSMV-*I and *PPSMV-II*) (Figure 2).

**TABLE 2:** Reaction of *Nicotiana* species to *Pigeonpea sterility mosaic virus* (*PPSMV*) by sap inoculation transmission.

S. no.	*Nicotiana* spp.	RT-PCR	
		*PPSMV-I*	*PPSMV-II*
1	*N. tabacum* cv. Xanthi	−	−
2	*N. tabacum* cv. Smyrna (turkish)	−	−
3	*N. benthamiana*	*+*	*+*
4	*N. clevelandii*	−	−
5	*N. glutinosa*	−	−
6	*N. rustica*	−	−
7	*N. sylvestris*	−	−
8	*N. obtusifolia*	−	−
9	*N. suaveolens*	−	−
10	*N. nudicaulis*	−	−
11	*N. repanda*	−	−

*+, positive; −, negative.*

When 16 cultivated crop species and 46 weed species were tested (Table 3) by leaf stapling method inoculation, *Phaseolus vulgaris* cvs. Top Crop, Bountiful, and Kintoki (F: Fabaceae) were infected with *PPSMV* but not supported for mite multiplication. The symptoms appeared as stunting, reduced leaf size, and mild crinkling. A weed host, *Chrozophora rottleri* (F: Euphorbiaceae) was also tested positive for *PPSMV* infection; however, there were no visual symptoms of virus infection or mites. A member of Fabaceae, *Macroptilium atropurpureum*, was found infested with a few mites but tested negative for *PPSMV*. Similar to the *Nicotiana* species, all the ELISA-positive samples when tested in RT-PCR were found infected with both the viruses (Figure 2). Though *P. vulgaris* cvs. Top Crop, Bountiful, Kintoki, and *C. rottleri* were found infected with the virus, they have not supported mite multiplication, and therefore, were not considered to be a potential source of inoculum for SMD.

**TABLE 3:** Reaction of different crop and weed species to mite inoculation transmission of *PPSMV*.

S. no.	Plant species	Mites/leaf*^a^*	ELISA	RT-PCR	
			
Crop species				*PPSMV I*	*PPSMV II*
1	*Arachis hypogaea*	−	−	*	*
2	*Capsicum annuum*	−	−	*	*
3	*Cicer arietinum*	−	−	*	*
4	*Dolichos lablab*	−	−	*	*
5	*Eleusine coracana* subsp. *Coracana*	−	−	*	*
6	*Glycine max*	−	−	*	*
7	*Gossypium hirsutum*	−	−	*	*
8	*Macrotyloma uniflorum*	−	−	*	*
9	*Pennisetum glaucum*	−	−	*	*
10	*Phaseolus vulgaris* cv. Bountiful	−	+	+	+
11	*Phaseolus vulgaris* cv. Kintoki	1	+	+	+
12	*Phaseolus vulgaris* cv. Top Crop	2	+	+	+
13	*Solanum lycopersicum*	−	−	*	*
14	*Sorghum bicolor*	−	−	*	*
15	*Vigna unguiculata* c-152	−	−	*	*
16	*Zea mays*	−	−	*	*

**S. no.**	**Plant species**	**Mites/leaf*^a^***	**ELISA**	**RT-PCR**	
			
**Weed species**				***PPSMV I***	***PPSMV II***

1	*Abelmoschus ficulneus*	−	−	*	*
2	*Abutilon indicum*	−	−	*	*
3	*Acanthospermum hispidum*	−	−	*	*
4	*Achyranthes aspera*	−	−	*	*
5	*Ageratum conyzoides*	−	−	*	*
6	*Alternanthera pungens*	−	−	*	*
7	*Amaranthus viridis*	−	−	*	*
8	*Argemone mexicana*	−	−	*	*
9	*Bidens biternata*	−	−	*	*
10	*Cardiospermum helicacabum*	−	−	*	*
11	*Cassia tora*	−	−	*	*
12	*Chenopodium amaranthicolor*	−	−	*	*
13	*Chenopodium album*	−	−	*	*
14	*Chloris barbata*	−	−	*	*
15	*Chromolaena odorata*	−	−	*	*
16	*Chrozophora rottleri*	−	+	+	+
17	*Crotalaria juncea*	−	−	*	*
18	*Cyperus rotundus*	−	−	*	*
19	*Cynodon dactylon*	−	−	*	*

**S. no.**	**Plant species**	**Mites/leaf*^a^***	**ELISA**	**RT-PCR**	
			
				***PPSMV I***	***PPSMV II***

20	*Datura stramonium*	−	−	*	*
21	*Eleusine coracana* subsp. *Africana*	−	−	*	*
22	*Euphorbia heterophylla*	−	−	*	*
23	*Euphorbia hirta*	−	−	*	*
24	*Hibiscus panduriformis*	−	−	*	*
25	*Lantana camara*	−	−	*	*
26	*Macroptilium atropurpureum*	2	−	*	*
27	*Malvastrum coromandelianum*	−	−	*	*
28	*Mimosa pudica*	−	−	*	*
29	*Parthenium hysterophorus*	−	−	*	*
30	*Phyllanthus niruri*	−	−	*	*
31	*Physalis floridana*	−	−	*	*
32	*Portulaca oleracea*	−	−	*	*
33	*Solanum xanthocarpum*	−	−	*	*
34	*Solanum nigrum*	−	−	*	*
35	*Synedrella nodiflora*	−	−	*	*
36	*Tridax procumbens*	−	−	*	*
37	*Xanthium strumarium*	−	−	*	*

^a^Average mites from three leaves. +, positive; −, negative; *, not tested.

**FIGURE 3:**
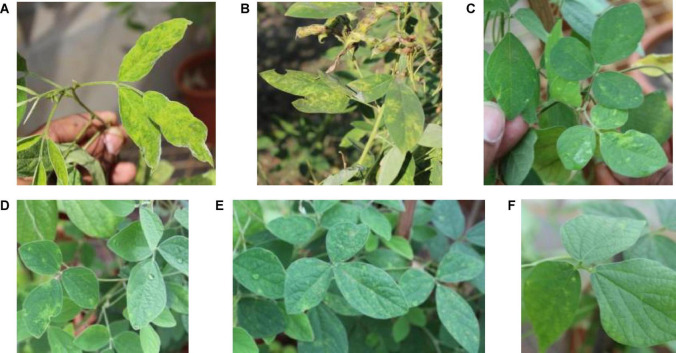
Typical sterility mosaic disease (SMD) symptoms on pigeonpea as yellow mosaic **(A)**, chlorotic spots **(B)**, mite-inoculated *PPSMV* infected wild *Cajanus* accessions exhibiting SMD symptoms **(C–F)**.

**FIGURE 4:**
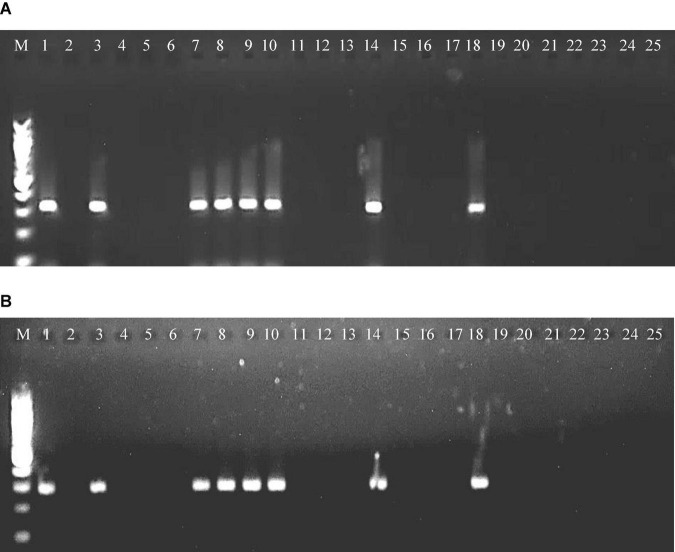
RT-PCR amplified product of RNA-3 segment of *PPSMV-I* **(A)** and *PPSMV-II* **(B)** from wild *Cajanus* accessions in 1.2% agarose gels. Standard 100-bp DNA marker (lane M), *Cajanus cajan* (lane 1), *C. cinereus* 15874 (lane 2), *C. lanceolatus* 15639 (lane 3), *C. marmoratus* 15651 (lane 4), *C. scarabaeoides* 15696 (lane 5), *C. scarabaeoides* 15711 (lane 6), *C. scarabaeoides* 15922 (lane 7), *C. scarabaeoides* 15683 (lane 8), *C. scarabaeoides* 15686 (lane 9), *C. platycarpus* 15666 (lane 10), *C. platycarpus* 15668 (lane 11), *C. platycarpus* 15671 (lane 12), *C. platycarpus* 15664 (lane 13), *C. platycarpus* 15661 (lane 14), *C. sericeus* 15760 (lane 15), *C. sericeus* 15762 (lane 16), *C. mollis* 15658 (lane 17), *C. reticulatus* 15675 (lane 18), *C. acutifolius* 15603 (lane 19), *C. acutifolius* 15611 (lane 20), *C. albicans* 15614 (lane 21), *C. albicans* 15620 (lane 22), *C. crassus* 15767 (lane 23), *C. crassus* 15768 (lane 24), and *C. confertiflorus* 15674 (lane 25).

Among the 24 accessions of 12 wild *Cajanus* species tested for *PPSMV* infection and mite infestation (Table 4), the accessions 15661, 15668, and 15671 of *Cajanus platycarpus* and 15683, 15686, and 15922 of *C. scarabaeoides* were found positive for both the virus and mite vector. So, in nature, these can act as potential sources of SMD inoculum multiplication, whereas accession 15666 of *C. platycarpus*, 15696 of *C. scarabaeoides*, and 15639 of *C. lanceolatus* were infected with the virus though no mites were observed on them. While accessions 15614 and 15620 of *Candida albicans* and 15874 of *Cajanus cinereus* supported mite multiplication, no infection of *PPSMV* was found. The rest of the *Cajanus* spp. accessions were neither supportive of mite multiplication nor *PPSMV* infection (Figure 3). The accessions that are tested positive in ELISA were confirmed for the infection of both the viruses in RT-PCR (Figure 4).

**TABLE 4:** Reaction of wild *Cajanus* species to *PPSMV* by mite inoculation transmission.

S. No.	Wild *Cajanus* spp.	Accession no.	No. of mites*^a^*	Type of symptom*^b^*	ELISA	RT-PCR	
						*PPSMV-I*	*PPSMV-II*
1	*C. platycarpus*	15666	−	NS	+	+	+
		15668	2	NS	−	−	−
		15671	1	NS	−	−	−
		15661	3	MM	+	+	+
		15664	−	NS	−	−	−
2	*C. scarabaeoides*	15696	−	NS	−	−	−
		15922	12	MM	+	+	+
		15683	2	SM	+	+	+
		15686	10	MM	+	+	+
		15711	−	NS	−	−	−
3	*C. sericeus*	15760	−	NS	−	−	−
		15762	−	NS	−	−	−
4	*C. acutifolius*	15603	−	NS	−	−	−
		15611	−	NS	−	−	−
5	*C. albicans*	15614	3	NS	−	−	−
		15620	2	NS	−	−	−
6	*C. mollis*	15658	−	NS	−	−	−
7	*C. crassus*	15767	−	NS	−	−	−
		15768	−	NS	−	−	−
8	*C. confertiflorus*	15674	−	NS	−	−	−
9	*C. lanceolatus*	15639	−	NS	**+**	**+**	**+**
10	*C. marmoratus*	15651	−	NS	−	−	−
11	*C. cinereus*	15874	12	NS	−	−	−
12	*C. reticulatus*	15675	−	NS	**+**	**+**	**+**
13	*C. cajan*	ICP-8863	34	SM	+	+	+

*^a^Mean count from three leaves/plant.*

*^b^NS, no symptom; MM, mild mosaic; SM, severe mosaic.*

*+, positive; −, negative.*

### Phylogenetic Analysis of *Pigeonpea sterility mosaic virus I* and *II* Nucleotide Sequences

**FIGURE 5:**
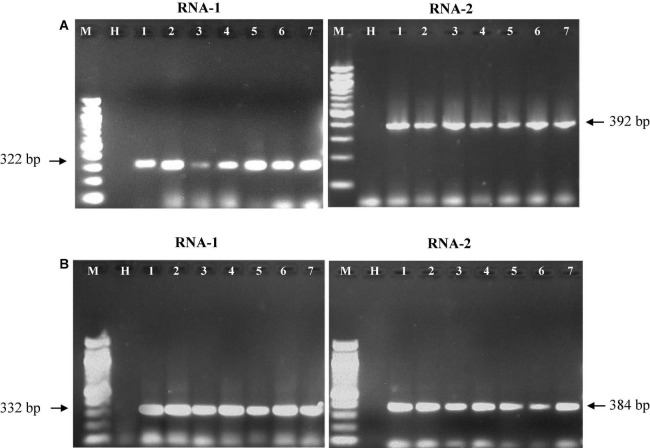
Resolution of RT-PCR products of RNA-1 and RNA-2 segments of *PPSMV-I* **(A)** and *PPSMV-II* **(B)** in 1.2% agarose gels. Standard 100-bp DNA marker (lane M), healthy pigeonpea leaf sample (lane H), lanes 1–7 are the SMD-affected pigeonpea samples from different geographical locations in southern India that were analyzed.

**FIGURE 6:**
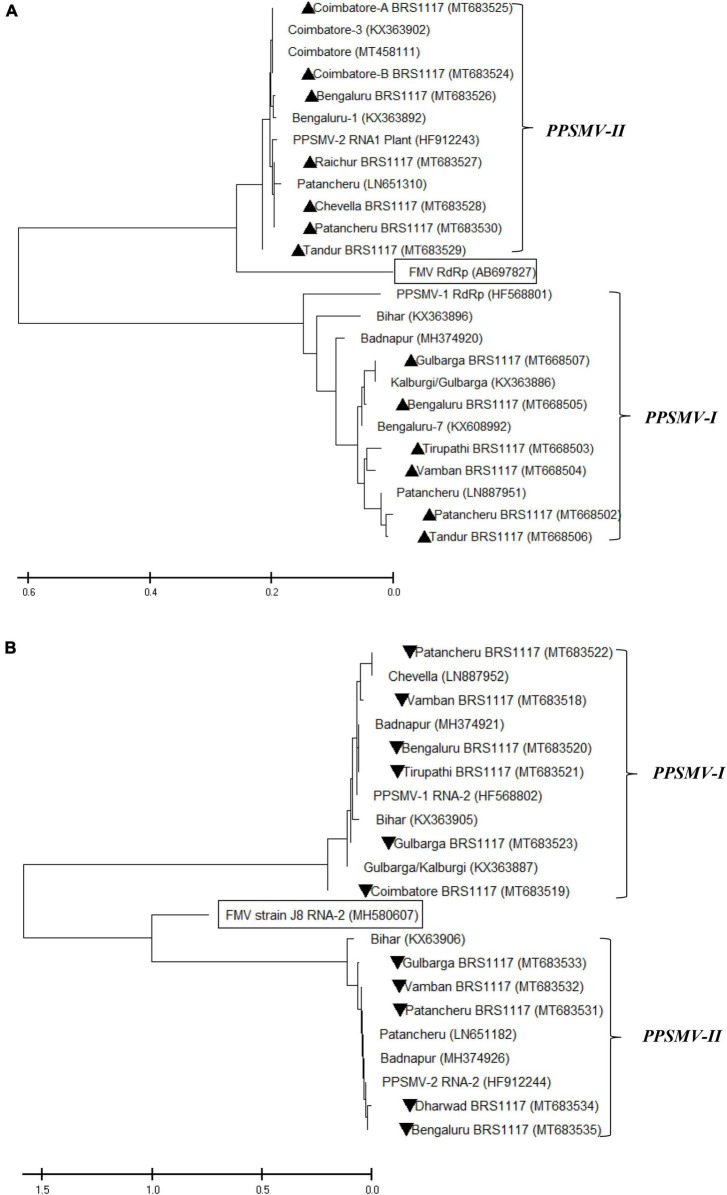
Phylogenetic analysis of nucleotide sequences of RNA-1 **(A)** and RNA-2 **(B)** of *PPSMV-I* and *PPSMV-II* along with available corresponding sequences. The Gene Bank accession numbers are given in parenthesis. The scale bar represents 0.2 and 0.5 substitutions per nucleotide position for RNA-1 and RNA-2 sequences, respectively. Symbols ▲ and ▼ represents virus isolates analyzed in the present study.

The oligonucleotide primers targeted for RNA-1 and RNA-2 of *PPSMV-I* and *PPSMV-II* resulted in amplicons of distinct sizes (Figure 5). The nucleotide sequences of RNA-1 when subjected to phylogenetic analysis along with available corresponding sequences, the isolates of *PPSMV-I* and *PPSMV-II*, formed two separate and distinct clusters (Figure 6). The isolates Coimbatore-A BRS1117, Coimbatore-B BRS1117, Bengaluru BRS1117, Raichur BRS1117, Patancheru BRS1117, and Chevella BRS1117 of *PPSMV-II* clustered into a distinct subcluster, and the isolate Tandur BRS1117 distinctly separated. The RNA-1 sequence of *Fig mosaic virus* (*FMV*) also clustered with *PPSMV-II* isolates, whereas the RNA-1 of *PPSMV-I* isolates Gulbarga BRS1117 and Bengaluru BRS1117 clustered together, and the isolates Tirupati BRS1117, Vamban BRS1117, Patancheru BRS1117, and Tandur BRS1117 clustered in another subcluster. Similarly, distinct clusters were formed when the RNA-2 sequences of *PPSMV-I* and *PPSMV-II* were subjected to the phylogenetic analysis in which the RNA-2 sequence of *PPSMV-I* isolate Coimbatore BRS1117 and *PPSMV-II* isolate Gulbarga BRS1117 were distinctly separated from the rest of the isolates. The RNA-2 sequence of *FMV* is also clustered with *PPSMV-II* isolates. The RNA-1 and RNA-2 sequences of *PPSMV-I* and *PPSMV-II* isolates were phylogenetically closer. However, RNA-1 and RNA-2 sequences of *PPSMV-II* isolates exhibited a close relationship with *FMV* than the *PPSMV-I*.

### Broad-Based Resistance to Sterility Mosaic Disease in Pigeonpea Genotypes

Screening of pigeonpea genotypes to SMD in different geographic locations revealed considerable variations in response to SMD infection (Table 5). Susceptible check (ICP 8863) showed a highly susceptible reaction (>40% incidence) in all three locations. The susceptible test genotypes exhibited typical SMD symptoms between 15 and 18 days after postinoculation (dpi). Among the test entries, ICPL-16086 and ICPL-16087 showed resistance reactions (<10% incidence) in all three locations, whereas genotypes ICPL-16078 and ICPL-16079 showed resistance reaction at the Bengaluru and Coimbatore locations, while at the Patancheru location showed moderate (10.1–20.0% incidence) and susceptible (20.1–40% incidence) reactions, respectively. The genotypes ICPL-16072, ICPL-16077, and ICPL-16083 expressed resistant reaction at the Coimbatore and Patancheru locations, but highly susceptible reaction at the Bengaluru location. ICPL-16050 and ICPL-16052 exhibited resistant reaction in Coimbatore, while they exhibited highly susceptible reaction at both the Bengaluru and Coimbatore locations.

**TABLE 5:** Screening of pigeonpea advanced breeding lines for their reaction to sterility mosaic disease (SMD) at different geographic locations during the rainy seasons of 2017/2018 and 2018/2019.

S. no.	Genotype	Days to symptom initiation	Bengaluru		Coimbatore		Patancheru		Overall avg. PDI	Overall reaction
		
			Avg.*^a^* per cent disease incidence (PDI)	Reaction*^b^*	Avg. PDI	Reaction	Avg. PDI	Reaction		
1	ICPL-16050	16–18	55.06	HS	0.00	R	40.54	HS	31.87	S
2	ICPL-16052	16–18	52.74	HS	8.33	R	51.23	HS	37.43	S
3	ICPL-16053	16–18	14.17	MR	19.62	MR	26.18	S	19.99	MR
4	ICPL-16054	17–19	17.65	MR	59.52	HS	61.32	HS	46.16	HS
5	ICPL-16058	17–19	42.86	HS	23.04	S	67.63	HS	44.51	HS
6	ICPL-16059	16–18	3.13	R	68.24	HS	43.06	HS	38.14	S
7	ICPL-16061	17–19	17.50	MR	12.14	MR	38.89	S	22.84	S
8	ICPL-16065	17–19	45.54	HS	64.38	HS	25.00	S	44.97	HS
9	ICPL-16067	15–17	56.25	HS	0.00	R	33.52	S	29.92	S
10	ICPL-16068	16–18	17.65	MR	0.00	R	59.79	HS	25.81	S
11	ICPL-16072	17–19	41.90	HS	2.94	R	2.38	R	15.74	MR
12	ICPL-16077	17–19	46.05	HS	0.00	R	0.00	R	15.35	MR
13	ICPL-16078	16–18	4.17	R	0.00	R	18.18	MR	7.45	R
14	ICPL-16079	18–20	0.00	R	0.00	R	30.30	S	10.10	MR
15	ICPL-16081	17–19	35.63	S	33.82	S	30.56	S	33.34	S
16	ICPL-16083	17–19	42.61	HS	0.00	R	5.26	R	15.96	MR
17	ICPL-16085	18–20	40.63	HS	10.83	MR	0.00	R	17.15	MR
**18**	**ICPL-16086**	**18–20**	**0.00**	**R**	**0.00**	**R**	**2.94**	**R**	**0.98**	**R**
**19**	**ICPL-16087**	**18–20**	**0.00**	**R**	**2.78**	**R**	**0.00**	**R**	**0.93**	**R**
20	ICPL-16088	14–16	86.15	HS	18.89	MR	0.00	R	35.01	S
21	ICP-8863*	13–15	91.67	HS	85.19	HS	96.30	HS	91.05	HS

*^a^Average percent disease incidence of SMD in 2017/2018 and 2018/2019.*

^b^R, resistant (≤10.00%); MR, moderately resistant (10.1–20.0%); S, susceptible (20.1–40.0%); HS = highly susceptible (>40.00%). *Indicate Highly susceptible genotype/check. Bold values indicated that, genotypes exhibited resistance reaction to SMD in all 3 locations tested.

## Discussion

Our study described the host range of *PPSMV-I* and *PPSMV-II*, the molecular relationship between them, and the source of resistance to SMD. The host range of *PPSMV* is narrow and confined to *Nicotiana benthamiana* through sap inoculation. The evidence of susceptibility of *N. benthamiana* to a wide range of plant viruses has been provided by Yang et al. (2004) as it has been linked to a naturally occurring mutation in an RNA-dependent RNA polymerase (RdRp) gene in the genome. The present study followed Kumar et al. (2002a; 2002b) and Manjunatha et al. (2018) who successfully transmitted *PPSMV* onto *N. benthamiana* and *Nicotiana clevelandii* by sap inoculation, but not on to the pigeonpea or any herbaceous hosts. Through mite inoculation, both the viruses can be successfully transmitted onto *Phaseolus vulgaris* cvs. Top Crop, Bountiful, Kintoki (F: Fabaceae) and *Chrozophora rottleri* (F: Euphorbiaceae), and was confirmed by the reports of Kulkarni et al. (2003a) and Kumar et al. (2004). However, there were contradicting observations of mite infestation on *Hibiscus panduriformis*, as in our study, neither the mite infestation nor *PPSMV* infection was found in both the field-collected samples as well as in artificially inoculated plants of *H. panduriformis*. Among the 24 accessions of 12 wild *Cajanus* species tested for the *PPSMV* infection, the accessions 15661, 15668, and 15671 of *C. platycarpus* and 15683, 15686, and 15922 of *C. scarabaeoides* were positive for both the viruses and supported mite multiplication, confirming earlier reports of Kulkarni et al. (2003b) and Kumar et al. (2005) that they can harbor the virus and vector and act as potential sources of inoculum in the field.

The study of the diversity of *PPSMV-I* and *PPSMV-II* associated with SMD of pigeonpea showed that these two emaraviruses are widespread across southern India. Analysis of sequence identity among the isolates of *PPSMV-I* and *PPSMV-II* indicated the presence of significant sequence variability. The RNA-1 and RNA-2 sequences of *PPSMV-II* isolates exhibited a close relationship with *FMV* than with *PPSMV-I*, and it is convincing and in agreement with the previous reports of Elbeaino et al. (2015); Kumar et al. (2017), Patil et al. (2017), and Sayiprathap et al. (2020) suggesting that these two emaraviruses infecting pigeonpea have followed two independent evolutionary paths. Patil et al. (2017) reported the prevalence of the *PPSMV-II* in the Coimbatore and Bengaluru locations. However, when we analyzed samples from different geographical locations in southern India, there is an existence of both the viruses in the Coimbatore and Bengaluru locations. This development of mixed infection over the years is possibly due to the spread of the virus to these locations by its mite vector, *A. cajani*. In nature, mites are the only means of transfer of SMD causal agent to pigeonpea and not through seed, pollen, or soil (Seth, 1962; Reddy et al., 1998; Kulkarni et al., 2002; Jones et al., 2004; Kumar et al., 2004; Patil and Kumar, 2015).

Host-plant resistance is the most viable and cost-effective option for the management of any viral disease. Though several researchers identified resistant sources to SMD in the past, most of their studies involved evaluation in one location with one isolate/strain (Nene, 1995). In contrast, our efforts led to the identification of two resistant genotypes such as ICPL-16086 and ICPL-16087 with broad-based resistance to SMD. In the present study, we also found high susceptibility of pigeonpea genotypes at the Bengaluru location, so the Bengaluru isolate could be considered as the severe strain in causing SMD in pigeonpea, and this was confirmed in an earlier report too (Nene et al., 1989). The variation in the disease reaction in different locations, attributed to different eriophyid mite vectors, was ruled out previously as Kumar et al. (2001b) reported that there is only one biotype present in India, which is transferring SMD to pigeonpea. So, the possible variation in our study is mainly due to virus variants, as Reddy et al. (1993) identified five distinct virus variants in India with different levels of virulence. There are conflicting reports about the genetics of resistance to SMD claiming both resistance and susceptibility to being dominant. Nevertheless, in most cases, susceptibility was shown to be dominant, and resistance is controlled by recessive genes (Singh and Vishwadhar, 2003). The resistance to SMD has been reported to be controlled by a single recessive gene (Srinivas et al., 1997) with oligogenic nature (Sharma et al., 1984; Gnanesh et al., 2011).

## Data Availability Statement

The datasets presented in this study can be found in online repositories. The names of the repository/repositories and accession number(s) can be found in the article/online.

## Author Contributions

BS conceptualized the work in discussion with AP and HS, executed the work in the field and glasshouse, and wrote the initial draft of the manuscript. VP, KJ, KS, and MS provided necessary inputs in designing the work plan. HR, ER, and LK provided all necessary support in the execution of the work in various locations. All authors contributed to the article and approved the submitted version.

## Conflict of Interest

The authors declare that the research was conducted in the absence of any commercial or financial relationships that could be construed as a potential conflict of interest.

## Publisher’s Note

All claims expressed in this article are solely those of the authors and do not necessarily represent those of their affiliated organizations, or those of the publisher, the editors and the reviewers. Any product that may be evaluated in this article, or claim that may be made by its manufacturer, is not guaranteed or endorsed by the publisher.

## Footnotes

1
https://blast.ncbi.nlm.nih.gov/Blast.cgi
